# Calcium imbalance drives organelle network collapse and immune remodeling: novel pathogenic mechanisms in MASLD progression

**DOI:** 10.3389/fimmu.2026.1879836

**Published:** 2026-07-01

**Authors:** Senping Xu, Ziyang Jiang, Qing Zhang, Jiawei Guo

**Affiliations:** 1 Department of Gastroenterology, The First Affiliated Hospital of Yangtze University, Jingzhou, Hubei, China; 2 Digestive Disease Research Institution of Yangtze University, Jingzhou, Hubei, China; 3 Clinical Medical College, Yangtze University, Jingzhou, Hubei, China; 4 Department of Pharmacology, School of Medicine, Yangtze University, Jingzhou, China; 5 Department of Neurology, Jingzhou Hospital Affiliated to Yangtze University, Jingzhou, China

**Keywords:** calcium signaling dysregulation, immune microenvironment remodeling, lipotoxicity, liver fibrosis, metabolic dysfunction-associated steatotic liver disease, mitochondrial dysfunction, NLRP3 inflammasome

## Abstract

Metabolic dysfunction-associated steatotic liver disease (MASLD) is initiated by ectopic lipid accumulation, but the precise mechanochemical transducers driving its progression to metabolic dysfunction-associated steatohepatitis (MASH) and fibrosis remain incompletely understood. This review comprehensively elucidates the central pathogenic role of intracellular calcium signaling dysregulation in MASLD. We detail how the metabolically toxic microenvironment induces pathological biophysical remodeling of lipid rafts and key calcium transporters across the plasma membrane (PM), endoplasmic reticulum (ER), and mitochondria. This pervasive transmembrane and inter-organellar calcium imbalance precipitates severe organelle network collapse, characterized by calcium depletion-driven ER stress, mitochondrial dysfunction, and the structural derangement of mitochondria-associated ER membranes (MAMs). Aberrant calcium fluxes function as critical secondary messengers that dictate hepatic immune microenvironment remodeling, at the cellular level driving Kupffer cell pro-inflammatory polarization, NLRP3 inflammasome assembly, and the amplification of damage-associated molecular patterns (DAMPs). These calcium-dependent immune-metabolic feedback loops synergistically trigger hepatic stellate cell (HSC) transdifferentiation and fibrogenesis. Finally, we highlight the latent calcium-regulatory mechanisms of current metabolic therapeutics and prospect the translational potential of targeted calcium modulators coupled with advanced nanodelivery systems, advocating for multi-targeted pharmacological strategies to arrest irreversible liver injury.

## 1 Introduction

In 2023, the international hepatology community redefined non-alcoholic fatty liver disease (NAFLD) as MASLD. Consequently, this paradigm shift underscores insulin resistance and metabolic dysfunction as core pathogenic drivers, reconceptualizing the hepatic phenotype as a localized manifestation of systemic metabolic derangement (1–3). Furthermore, MASLD pathogenesis intrinsically intertwines with central adiposity, dyslipidemia, and disrupted energy homeostasis (4, 5). During its protracted and insidious natural history, isolated steatosis can transition into MASH. Ultimately, persistent inflammation and parenchymal degeneration silently drive progression toward decompensated cirrhosis or hepatocellular carcinoma (HCC) (6–8).

Given this complex pathogenesis, the traditional ‘two-hit’ hypothesis has been superseded by a ‘multiple-hit’ model encompassing lipotoxicity, ER stress, and immune dysregulation (1, 9). Mechanistically, exogenous toxic lipids, including FFAs and cholesterol, precipitate profound ultrastructural alterations and plasticity remodeling across hepatic parenchymal and non-parenchymal cells, driving disease progression (10, 11). However, while lifestyle modifications and weight reduction can reverse early-stage MASLD, sustaining long-term clinical efficacy remains exceptionally challenging (12). Therefore, identifying the precise molecular hubs that irreversibly translate a dysmetabolic microenvironment into massive organellar collapse and tissue injury constitutes a paramount research priority.

Among emerging pathogenic mechanisms, dysregulated calcium signaling constitutes a pivotal node resolving this molecular conundrum. As a fundamental second messenger, aberrant calcium fluxes orchestrate the initiation and progression of steatotic liver disease (13, 14). Consequently, lipid overload and toxic metabolites incite severe lipotoxicity, disrupting calcium homeostasis to precipitate cascading ER stress and mitochondrial oxidative stress (mtROS) (15, 16). Importantly, the synergistic disruption of calcium signaling and autophagy exacerbates obesity-induced lipotoxicity, propelling MASLD toward end-stage hepatic disease (17, 18).

We hypothesize that dysregulated calcium signaling acts as a central nexus linking lipotoxicity, organellar stress, and hepatic immune dysfunction. Accordingly, this review delineates the lipotoxic microenvironment and early mechanosensory cellular disruptions. Furthermore, we dissect the pathological remodeling of key calcium transporters, encompassing PM influx, ER release, and mitochondrial uptake during MASLD progression. Subsequently, we elucidate how aberrant calcium fluxes precipitate organellar structural collapse. Consequently, this damage reshapes the hepatic immune microenvironment, driving downstream HSC activation and fibrogenesis. Finally, we evaluate the translational potential of pharmacological interventions targeting calcium-immune networks to provide precision therapeutic strategies arresting MASLD progression.

## 2 Early MASLD and the metabolically toxic microenvironment

### 2.1 Intrahepatic accumulation of lipogenic and glucogenic molecules

**Figure 1:**
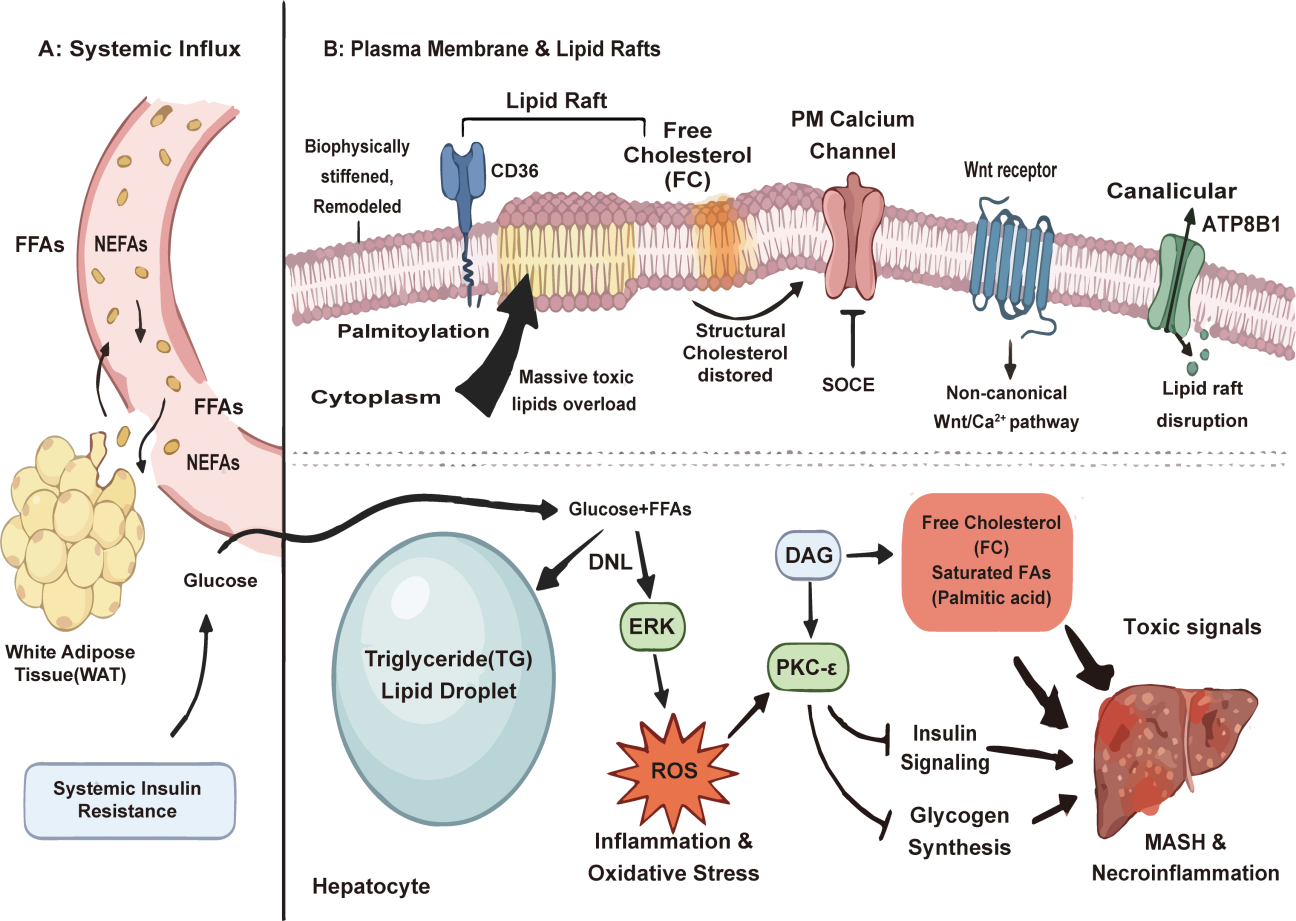
Mechanisms of Early MASLD: Metabolically Toxic Microenvironment and Lipid Raft Disruption. Systemic metabolic dysregulation drives massive influx of FFAs, NEFAs, and glucose into hepatocytes. **(A)** Intracellular Lipotoxicity: The uncoupling of lipid metabolism leads to the accumulation of TG into droplets. However, toxic mediators drive disease progression: excessive DAG activates PKC-ϵ, which blunts insulin signaling and abolishes glycogen synthesis. Concurrently, DNL and ERK activation fuel oxidative stress. Catastrophic accumulation of FC and saturated fatty acids triggers necroinflammation, driving the transition to MASH. **(B)** Membrane Microstructure Alterations: Lipotoxicity induces biophysical stiffening of the PM. Palmitoylation rigidly anchors CD36 within lipid rafts, amplifying toxic lipid uptake. FC overload alters membrane thickness, structurally distorting PM calcium channels and severely impairing SOCE. Additionally, membrane remodeling skews the Wnt cascade toward the non-canonical Wnt/Ca^2+^ pathway, while defects in ATP8B1 dismantle the canalicular lipid bilayer, collectively initiating early signal transduction failures and organellar collapse. DAG, Diacylglycerol; DNL, De novo lipogenesis; ERK, Extracellular signal-regulated kinase; FC, Free cholesterol; FFAs, Free fatty acids; MASH, Metabolic dysfunction-associated steatohepatitis; MASLD, Metabolic dysfunction-associated steatotic liver disease; NEFAs, Non-esterified fatty acids; PKC-ϵ, Protein kinase C ϵ; PM, Plasma membrane; ROS, Reactive oxygen species; SOCE, Store-operated calcium entry; TG, Triglyceride; WAT, Whiteadipose tissue.

Pathological ectopic lipid accumulation initiates MASLD. Physiologically, white adipose tissue (WAT) and the liver coordinate systemic energy homeostasis. However, systemic metabolic dysregulation, characterized by central adiposity and insulin resistance, disrupts adipose lipid storage. Therefore, massive quantities of free fatty acids (FFAs) flood the circulation, driving uncontrolled hepatic influx (19, 20). Peripheral insulin resistance escalates the hepatic delivery of lipogenic substrates, encompassing excess glucose and non-esterified fatty acids (NEFAs), thereby overwhelming hepatocellular metabolic capacity (1, 21). In addition, concurrent hyperglycemia and elevated FFAs fuel hepatic *de novo* lipogenesis (DNL) and activate stress kinases, including ERK. Taken together, this synergy precipitates a lipotoxic microenvironment defined by early-stage inflammation and oxidative stress (**Figure 1**) (7, 22).

Profound exogenous lipid and carbohydrate influx abolishes hepatic metabolic homeostasis. Initially, hepatocytes mount a compensatory defense, sequestering toxic lipids into inert triglyceride (TG) droplets to manifest as isolated steatosis (19, 23). However, persistent uncoupling of fatty acid uptake, DNL, lipid oxidation, and very-low-density lipoprotein (VLDL) secretion precipitates extreme hepatic lipid overload (24, 25). Furthermore, pathological upregulation of hepatocellular lipid transporters, prominently CD36, exacerbates long-chain fatty acid influx and lipotoxic deposition (26, 27). Accordingly, excessive diacylglycerol (DAG) accumulation aberrantly activates PKC-ϵ, which blunts insulin signaling and abolishes glycogen synthesis. This triggers primary hepatic insulin resistance, driving a self-perpetuating pathogenic cycle (**Figure 1**) (21).

Beyond TG accumulation, structural and proportional imbalances of specific lipid mediators exert profound lipotoxicity, driving MASLD progression to MASH. In particular, an aberrant saturated to unsaturated fatty acid ratio, notably involving palmitic acid, directly inflicts severe hepatocellular toxicity and disrupts intracellular neutral lipid homeostasis (28–30). Beyond this, dysregulated cholesterol metabolism constitutes a core pathogenic driver. Uncoupling of cholesterol *de novo* synthesis, intestinal absorption, and systemic clearance precipitates catastrophic intrahepatic free cholesterol (FC) accumulation (31, 32). In contrast to isolated lipid loading, FC overload functions as a potent toxic signal, directly mediating hepatocellular necroinflammation and microenvironmental degradation. Consequently, this dictates the critical transition from isolated steatosis to MASH, fibrogenesis, and HCC (**Figure 1**) (33–35). Overall, this extreme metabolic overload, propelled by pleiotropic toxic metabolites, plunges hepatocytes into uncompensated adaptive failure, establishing the primary mechanistic link between systemic metabolic dysregulation and downstream organellar collapse (36, 37).

### 2.2 Alterations in lipid raft microstructure and early signal disruption

During MASLD pathogenesis, lipotoxicity-induced biophysical alterations in hepatocyte membranes represent a critical pathogenic nexus. Specifically, saturated fatty acid-driven lipidomic remodeling severely compromises membrane fluidity and bilayer integrity. Therefore, this structural degradation destabilizes membrane-associated proteins and calcium signaling networks (38, 39). Furthermore, defects in critical lipid flippases, notably ATP8B1, dismantle the canalicular lipid bilayer, establishing a foundational structure.

al lesion that initiates early signal transduction failures (40). Ultimately, this biophysical stiffening abrogates normal transmembrane signaling, severely compromising cellular metabolic stress sensing and ionic homeostasis.

Importantly, lipotoxicity biophysically dysregulates ion channels, notably calcium channels, via PM lipid raft remodeling. As a fundamental microdomain constituent, cholesterol orchestrates lipid raft assembly, hormone synthesis, and transmembrane signal transduction, encompassing the Hedgehog cascade (31). However, FC overload aberrantly alters PM tension and thickness. Accordingly, these biophysical perturbations distort the spatial conformation of crucial PM calcium channels. This structural distortion severely impairs store-operated calcium entry function (**Figure 1**) (41).

As a result, lipid raft biophysical perturbations profoundly alter the membrane localization and early signaling of other critical receptors. Specifically, lipotoxic milieus hyperactivate site-specific palmitoylation, aberrantly anchoring fatty acid transporters, notably CD36, within PM lipid rafts. Consequently, this rigid stabilization amplifies toxic lipid uptake and derails early signaling cascades (**Figure 1**) (27). Furthermore, pathological membrane remodeling prominently skews spatially dependent intercellular communication, notably the Wnt cascade, toward the non-canonical Wnt/Ca^2+^ pathway (**Figure 1**). This diversion aberrantly modulates intracellular calcium fluxes, directly dictating cellular fate (42). Overall, membrane biophysical degradation transcends mere physical injury, functioning as a central mechanochemical transducer that converts lipotoxicity into severe downstream calcium signaling aberrations and organellar collapse.

## 3 Pathological remodeling of key calcium transporters in MASLD

### 3.1 Overexpression of the PM calcium influx system

**Figure 2:**
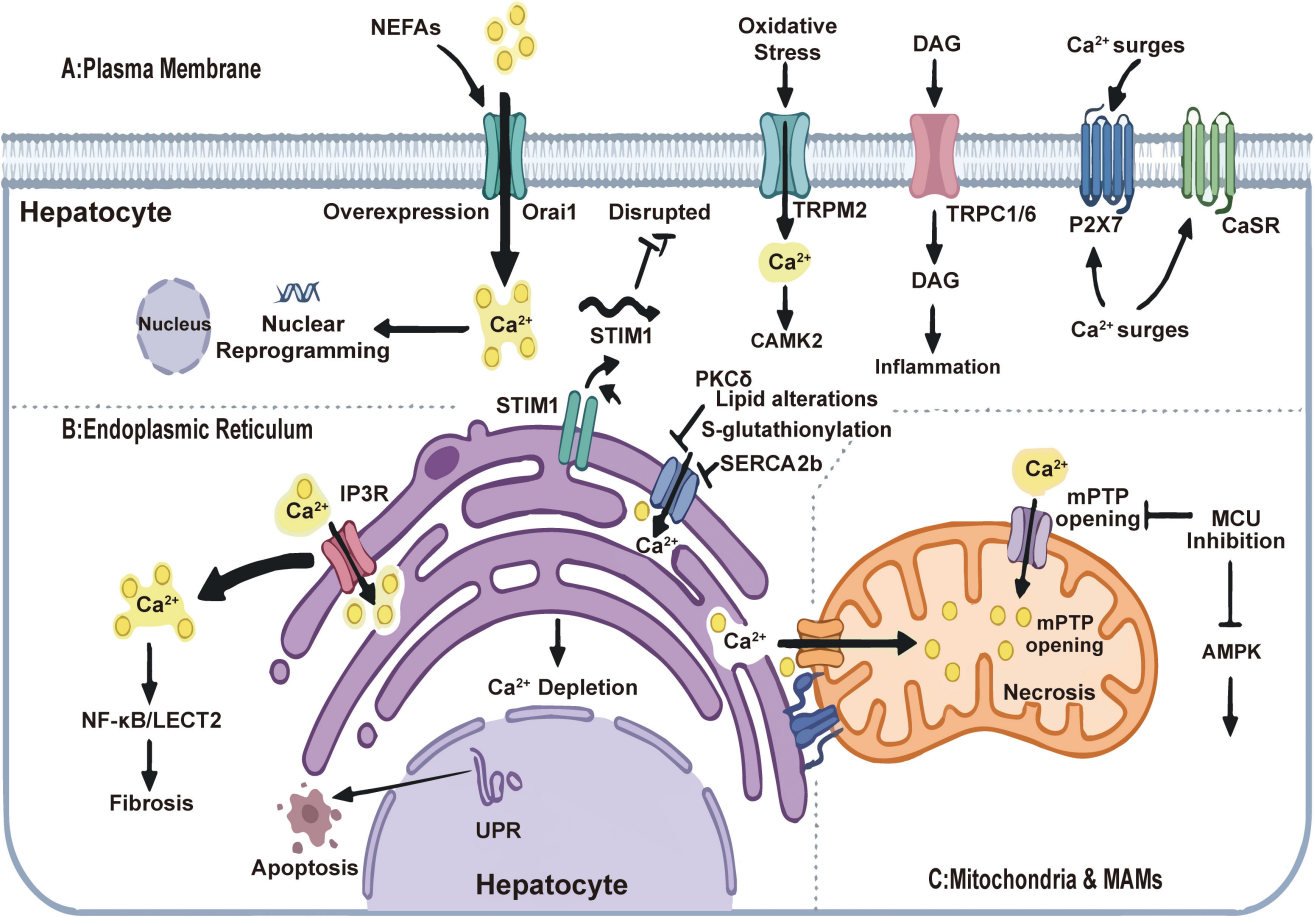
Pathological remodeling of calcium transport networks in MASLD. The lipotoxic microenvironment in MASLD profoundly disrupts hepatocellular calcium homeostasis across three major subcellular domains. **(A)** Plasma Membrane Overload: Lipids and oxidative stress pathologically hyperactivate PM calcium channels. Upregulation of Orai1 and aberrant activation of TRP channels alongside purinergic receptors drive massive cytosolic calcium influx, initiating CAMK2 activation, nuclear reprogramming, and inflammation. **(B)** Endoplasmic Reticulum Depletion: Intracellularly, lipotoxicity severely sensitizes IP3R channels, causing persistent calcium leakage into the cytoplasm, which triggers the Ca^2+^/NF-κB/LECT2 fibrogenic cascade. Concurrently, SERCA2b pumps are drastically inhibited by PKCδ, altered membrane lipids, and S-glutathionylation. This combined leakage and failed reuptake precipitate severe ER luminal calcium depletion, leading to the UPR and apoptosis. **(C)** Mitochondrial Calcium Shift: Through enhanced MAMs, leaked ER calcium is funneled directly into mitochondria via upregulated MCU complexes. This mitochondrial matrix calcium overload dissipates the membrane potential and forces mPTP opening, culminating in necrosis. Paradoxically, complete MCU ablation dephosphorylates AMPK, exacerbating basal lipid accumulation, highlighting the strict necessity for MCU homeostatic balance. AMPK, AMP-activated protein kinase; CAMK2, Calcium/calmodulin-dependent protein kinase II; CaSR, Calcium-sensing receptor; DAG, Diacylglycerol; ER, Endoplasmic reticulum; IP3R, Inositol 1, 4,5-trisphosphate receptor; LECT2, Leukocyte cell-derived chemotaxin 2; MAMs, Mitochondria-associated membranes; MASLD, Metabolic dysfunction-associated steatotic liver disease; MCU, Mitochondrial calcium uniporter; mPTP, Mitochondrial permeability transition pore; NEFAs, Non-esterified fatty acids; NF-κB, Nuclear factor kappa B; PKCδ, Protein kinase C δ; SERCA2b, Sarco/endoplasmic reticulum calcium-ATPase 2b; SOCE, Store-operated calcium entry; STIM1, Stromal interaction molecule 1; TRP, Transient receptor potential; UPR, Unfolded protein response.

SOCE constitutes the predominant plasma membrane calcium influx mechanism in non-excitable cells, maintaining basal homeostasis (43, 44). Physiologically, STIM1 functions as an ER calcium sensor. Upon detecting luminal depletion, STIM1 translocates to ER-PM (PM) junctions, recruiting and activating the highly selective PM Orai1 channel to replenish intracellular calcium stores (41, 45, 46). However, the lipotoxic MASLD microenvironment severely disrupts this adaptive feedback, precipitating hepatic metabolic dysfunction (47). Mechanistically, lipid metabolism dysregulation and subsequent membrane domain cholesterol depletion alter Orai1 diffusion and clustering, potentially triggering aberrant channel internalization (41). Concurrently, hepatocellular Orai1 expression demonstrates marked sensitivity to NEFA accumulation. Therefore, NEFA overload acutely upregulates surface Orai1 expression, aberrantly amplifying calcium-dependent DNL (48–50). Under persistent lipotoxicity, hyperactivated SOCE drives massive cytoplasmic calcium influx along a profound electrochemical gradient. This sustained calcium overload translocates downstream transcription factors into the nucleus, initiating pathological reprogramming (**Figure 2**) (51–54). Although isolated steatosis may transiently inhibit SOCE via PKC-dependent pathways (55), the global kinetic disruption of the SOCE network remains the paramount driver of PM calcium signaling collapse (14).

Beyond the classical SOCE pathway, aberrant expression and functional remodeling of the transient receptor potential (TRP) ion channel family, widely expressed in hepatic tissue, profoundly contribute to lipotoxicity-mediated metabolic injury (56–58). Specifically, TRPM2, an oxidative stress-sensitive, calcium-permeable non-selective cation channel, exerts a critical pathogenic role in hepatic metabolic dysregulation and tissue injury (59, 60). Evidence from murine knockout models and human hepatocyte cell lines demonstrates that in the lipotoxic MASLD milieu, oxidative stress directly precipitates TRPM2-mediated PM calcium influx. Consequently, this aberrant upstream signal hyperactivates calcium/calmodulin-dependent protein kinase II (CAMK2), initiating downstream hepatocellular death cascades and driving malignant transformation toward primary HCC (**Figure 2**) (61–63). Concurrently, receptor-operated calcium channels (ROCs) and select SOCEs predominantly assemble as heteromeric TRPC complexes, notably TRPC1 and TRPC6. These channels respond to store depletion and are directly activated by G protein-coupled receptor-derived lipid second messengers, prominently DAG.This rapid pathogenic calcium influx exacerbates hepatic inflammation and fibrogenesis (**Figure 2**) (51, 64). Meanwhile, TRPM7, possessing dual ion channel and kinase functionalities, augments calcium influx upon direct CaM regulation, thereby amplifying reactive oxygen species (ROS) generation during cellular stress (65). Conversely, TRP family activation is not uniformly deleterious. For instance, observations in murine models show that TRPV4 activation in liver sinusoidal endothelial cells (LSECs) promotes endothelial nitric oxide synthase (eNOS) phosphorylation and release, exerting a hepatoprotective effect against oxidative toxicity during early-stage MASLD (66).

The profound dysregulation of the PM calcium influx system is exacerbated by synergistic perturbations across diverse channels and receptors. Experimental evidence indicates, the definitive hepatocellular expression of calcium-sensing receptors (CaSR) challenges the classical paradigm of the liver as a non-calcium-metabolizing organ, critically mediating lipid metabolism under lipotoxic conditions (67). In addition, hepatic metabolic homeostasis is directly modulated by purinergic receptors, notably the ionotropic P2X7 and select metabotropic P2Y subtypes. Upon lipotoxic stimulation, *in vitro* studies demonstrate that these receptors precipitate aberrant intracellular calcium transients (**Figure 2**). Accordingly, these transients synergize with non-canonical cascades, prominently the Wnt/Ca^2+^ pathway, to trigger calcium surges that severely abrogate hepatocellular function (42, 68). Taken together, the pathological hyperactivation of pleiotropic PM ion channels, coupled with dysregulated homeostatic factors including the novel microenvironmental downregulator SLC10A7, orchestrates an uncontrolled transmembrane calcium influx network (52, 69). Mechanistically, this systemic calcium signaling imbalance drives progressive hepatic injury and severe metabolic deterioration (33). Conversely, pharmacological blockade of aberrant calcium influx, notably utilizing verapamil, effectively restores intracellular calcium homeostasis and significantly mitigates lipotoxic injury in diet-induced obese murine models and human hepatoma cell cultures (17).

### 3.2 Imbalance in ER calcium release and uptake

As the principal intracellular calcium reservoir, ER maintains a steep luminal calcium gradient, critical for fundamental lipid homeostasis and proper metabolic protein folding, notably proinsulin. These changes, the disruption of ER calcium homeostasis constitutes a central pathogenic nexus linking lipotoxicity to the unfolded protein response (UPR) (28, 70). During MASLD progression, hepatocellular ER-resident inositol 1,4,5-trisphosphate receptor (IP3R) channels undergo profound pathological remodeling. Specifically, the type 2 receptor, ITPR2, the predominant mediator of hepatic intracellular calcium release, sustains severe kinetic impairment under lipotoxic stress. This impairment precipitates a catastrophic disruption of stimulus-responsive nuclear calcium signaling (71, 72). Experimental cell culture studies suggest the lipotoxic milieu induces extreme IP3R sensitization, driving the uncontrolled and persistent leakage of luminal calcium into the cytoplasm (36, 39). This aberrant calcium efflux disrupts basal calcium release kinetics and hyperactivates the type 3 receptor, ITPR3. Studies in murine models reveal that this sequential activation triggers the Ca^2+^/NF-κB/LECT2 signaling cascade, inflicting lethal hepatocellular injury and potently driving downstream fibrogenesis (**Figure 2**) (73, 74).

Concomitant with IP3R hypersensitization-induced calcium leakage, the functional collapse of the sarco/endoplasmic reticulum calcium ATPase (SERCA) system, responsible for cytoplasmic calcium reuptake, constitutes a parallel catastrophic event. In obesity and lipid overload models, predominant isoforms, notably hepatic SERCA2b, undergo precipitous transcriptional and translational downregulation. As a result, this depletion drastically attenuates the endogenous ER calcium storage capacity (75–77). The mechanisms driving SERCA impairment are profoundly pleiotropic. In particular, evidence from murine models and *in vitro* assays indicates that pathological shifts in ER membrane lipid composition biophysically constrain SERCA conformational dynamics (78). Furthermore, aberrant free fatty acid accumulation hyperactivates the PKCδ cascade, exerting a potent direct inhibition on SERCA pump activity (79). Concurrently, chronic oxidative stress propelled by metabolic dysregulation biochemically impairs SERCA via aberrant post-translational modifications, prominently peroxynitrite-mediated S-glutathionylation (22). Eventually, dysregulated expression of regulatory micropeptides, notably PLN and DWORF, alongside auxiliary proteins including Nwd1, deprives SERCA of the vital structural scaffolding requisite for precisely orchestrated calcium pumping (80, 81).

The synergistic detriment of IP3R-mediated pathological calcium leakage and SERCA pump failure precipitates rapid ER calcium depletion. Consequently, this depletion profoundly disrupts the intracellular lipid metabolic network and constitutes an absolute prerequisite for severe UPR and apoptosis induction (**Figure 2**) (13, 16, 17). This mechanistic derangement in ER calcium cycling underpins the core molecular pathogenesis in experimental steatosis models, notably thapsigargin-induced human induced pluripotent stem cell-derived hepatocyte (hiPSC-Hep) platforms (28). Ultimately, therapeutically targeting this cyclic imbalance demonstrates profound translational potential. Experimental evidence indicates, pharmacological blockade of aberrant IP3R leakage utilizing dantrolene, targeted PKCδ silencing, AMPK activation via Maresin 1, or SERCA agonism employing Urolithin A, effectively replenishes the ER calcium reservoir. Collectively, these interventions robustly reverse organellar stress and lipid overload-driven metabolic dysfunction (30, 82–85).

### 3.3 The pathogenic shift of the mitochondrial calcium unidirectional transport complex

MCU mediates cytosolic calcium entry into the mitochondrial matrix, crucially regulating hepatocyte energy metabolism and lipid homeostasis (38, 86). During MASLD progression, MCU function and its associated calcium signaling network undergo a pathogenic shift. Multiple studies demonstrate significantly upregulated MCU expression in human and murine MASH models (87). Under lipid overload and persistent ER stress, aberrant MAM activation establishes a direct conduit for ER-to-mitochondria calcium flux. Therefore, this disrupts organellar compartmentalization, driving massive calcium influx into the mitochondrial matrix via the MCU (88–90). Notably, even during early latent stress from prolonged hyperglycemia lacking significant cytosolic free calcium fluctuations, MCU mediates a 49% increase in aberrant mitochondrial calcium uptake (91). Importantly, without hepatoprotective factors maintaining MAM integrity, notably hepatic stimulant-mediated SERCA preservation, this detrimental inter-organellar calcium transfer remains uninhibited (92).

Subsequently, MCU-mediated matrix calcium overload disrupts oxidative respiration, precipitating hepatocyte injury. Driven by upstream MCU1-dependent metabolic regulation involving mitochondrial pyruvate metabolism and aberrant fatty acid flux (93), sustained calcium overload dissipates the ΔΨm. This irreversibly triggers pathological mitochondrial permeability transition pore (mPTP) opening, forming a critical intersection linking necrosis and apoptosis (**Figure 2**) (65, 89).

However, MCU regulation within lipid metabolism presents a profound paradox. Studies utilizing hepatocyte-specific MCU knockout mice and adenovirus-mediated GCaMP6 calcium probes demonstrate that merely inhibiting MCU-mediated calcium uptake fails to reverse lipotoxicity. Instead, this inhibition severely impairs basal hepatic lipid metabolism via PP4-dependent AMPK dephosphorylation, identifying AMPK as a core downstream effector (**Figure 2**) (94). Moreover, prolonged exposure to metabolic toxicity diets exacerbates hepatomegaly and steatosis in MCU-deficient mice (95). This process, rather than exclusively driving pathogenic calcium overconsumption, MCU crucially balances basal lipid homeostasis against lethal calcium overload. Therefore, any bidirectional dysregulation of MCU activity inherently triggers mitochondrial dysfunction.

## 4 Calcium-driven organelle network stress and structural collapse

### 4.1 Severe ER driven by calcium depletion

High calcium concentrations within the ER lumen provide the biochemical foundation for maintaining protein folding and processing. In this microenvironment, calcium serves as an essential cofactor to maintain the catalytic conformations of molecular chaperones, predominantly calnexin and calreticulin (84). However, under metabolic and lipotoxic stress, aberrant lipid accumulation and free fatty acid overload rapidly deplete endogenous ER calcium reserves (75, 78). As a result, this sustained transmembrane calcium imbalance impairs chaperone function, precipitating a massive accumulation of misfolded proteins (39, 79). Therefore, calcium depletion-driven structural instability acts as the primary upstream mechanism disrupting ER homeostasis and triggering severe ER stress (16, 96).

**Figure 3:**
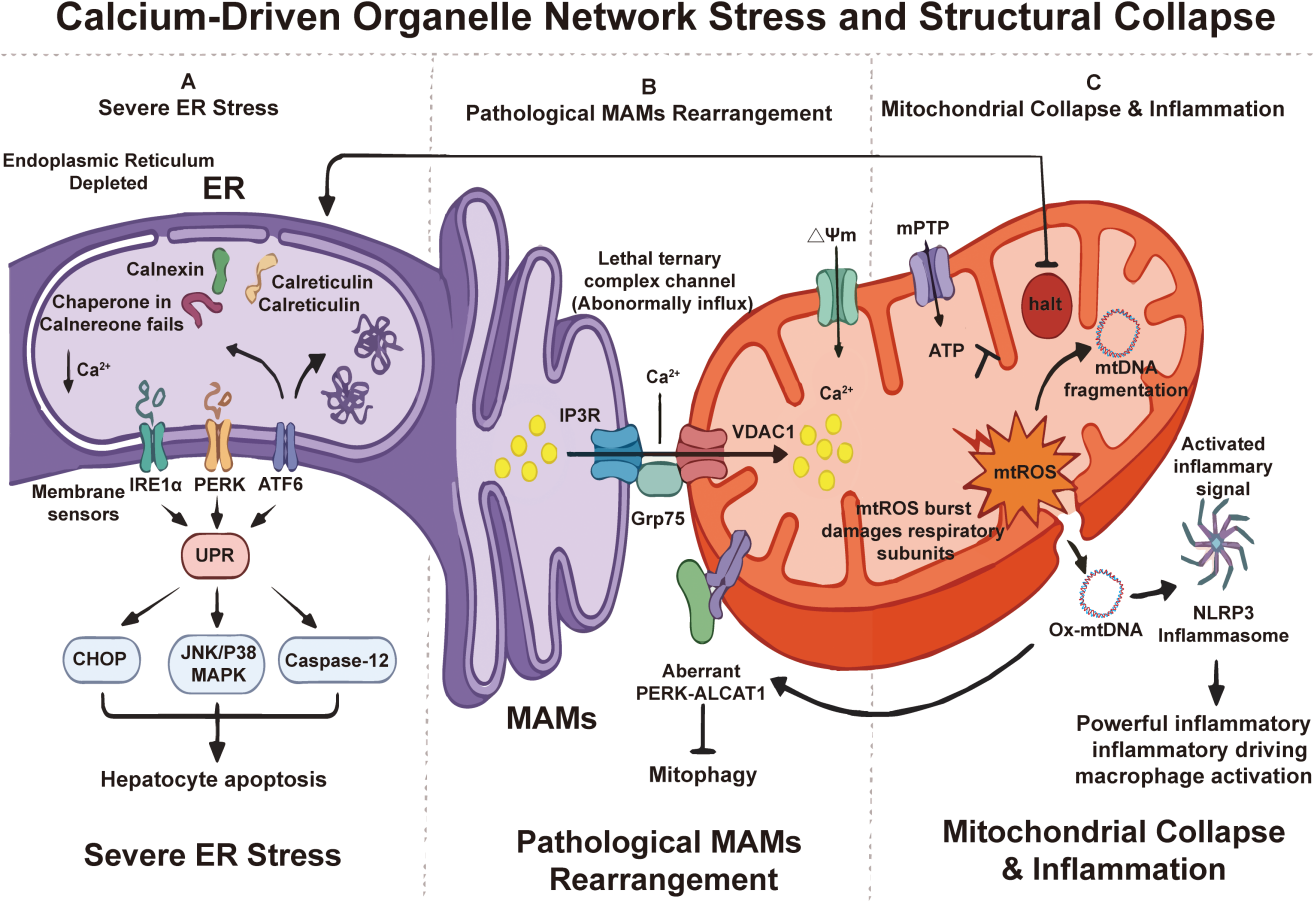
Calcium-driven organelle network stress and structural collapse. The lipotoxic microenvironment induces a catastrophic inter-organellar collapse mediated by aberrant calcium signaling. **(A)** Severe ER Stress: Transmembrane calcium depletion inside the ER inactivates chaperones, resulting in massive accumulation of misfolded proteins. This hyperactivates the UPR sensors, steering the cell toward a pro-apoptotic trajectory via CHOP, JNK/P38 MAPK, and Caspase-12 cascades. **(B)** Pathological MAMs Rearrangement: Lipid overload establishes abnormally tight physical contacts at MAMs. The IP3R-Grp75-VDAC1 ternary complex hyperactivates, creating a lethal conduit for massive ER-to-mitochondria calcium transfer, while aberrant PERK-ALCAT1 complexes impair protective mitophagy. **(C)** Mitochondrial Dysfunction & mtROS Burst: Matrix calcium overload triggers persistent mPTP opening, dissipating the membrane potential and halting ATP production. The resulting burst of mtROS damages the respiratory chain and fragments mtDNA. The subsequent cytosolic release of Ox-mtDNA, alongside ROS, potently activates the NLRP3 inflammasome, driving macrophage activation, severe hepatic inflammation, and irreversible hepatocyte lipoapoptosis. ALCAT1, Acyl-CoA, lysocardiolipin acyltransferase 1; ATF6, Activating transcription factor 6; ATP, Adenosine triphosphate; CHOP, C/EBP homologous protein; ER, Endoplasmic reticulum; Grp75, Glucose-regulated protein 75; IP3R, Inositol 1, 4,5-trisphosphate receptor; IRE1α, Inositol-requiring enzyme 1 α; JNK, c-Jun N-terminal kinase; MAMs, Mitochondria-associated membranes; MAPK, Mitogen-activated protein kinase; mPTP, Mitochondrial permeability transition pore; mtDNA, Mitochondrial DNA; mtROS, Mitochondrial reactive oxygen species; NLRP3, NLR family pyrin domain containing 3; Ox-mtDNA, Oxidized mitochondrial DNA; PERK, Protein kinase RNA-like endoplasmic reticulum kinase; ROS, Reactive oxygen species; UPR, Unfolded protein response; VDAC1, Voltage-dependent anion channel 1; ΔΨm, Mitochondrial membrane potential.

In response to accumulated misfolded proteins, hepatocytes activate the UPR. This signaling cascade utilizes three transmembrane sensor pathways, specifically IRE1α, PERK, and ATF6, to restore proteostasis by attenuating global protein synthesis and upregulating chaperones (96, 97). Nevertheless, the intensity and duration of ER stress dictate the cellular fate between homeostasis and apoptosis (98). In a persistent lipotoxic microenvironment, irreversible calcium depletion overwhelms chaperone repair capacity, driving the UPR toward a pro-apoptotic trajectory (28, 70). Subsequently, persistently activated PERK and IRE1α pathways upregulate the critical pro-apoptotic factor CHOP (36). Furthermore, this stress response synergistically activates the calcium-dependent JNK/P38 MAPK kinase cascade alongside the Calpain/Caspase-12 and Bax/Caspase apoptotic axes, ultimately executing irreversible hepatocyte apoptosis (**Figure 3**) (99, 100).

Severe ER stress driven by calcium pool depletion crucially mediates the pathogenesis of NAFLD and its progression to MASH. Experimental evidence indicates that ER quality control failure amplifies pathological hepatocyte lipid accumulation via hyperactive UPR signaling. Observations from these cell culture systems and broader murine models suggest that this multiorganellar dysfunction accelerates the transition from simple hepatic steatosis to diffuse inflammation, metabolic collapse, autophagy impairment, and widespread liver fibrosis (7, 28, 101).

### 4.2 Mitochondrial dysfunction and mtROS burst induced by calcium overload

Cytosolic-mitochondrial calcium flux is tightly regulated in healthy hepatocytes; however, lipid overload and metabolic stress disrupt this homeostasis. Accumulated FFAs and lipotoxic derivatives impair MAMs; studies have shown, *in vitro* and murine models have demonstrated that they alter inositol 1,4,5-trisphosphate receptor-voltage-dependent anion channel 1 (IP3R-VDAC1) coupling. This drives massive calcium influx from the ER into the mitochondrial matrix (25, 102). Consequently, this inter-organelle calcium imbalance propagates endoplasmic reticulum stress to the mitochondria, precipitating irreversible organellar damage (16, 38). Subsequent matrix calcium overload collapses ionic homeostasis and osmotic pressure, triggering sustained mPTP opening. This pathological pore activation dissipates the inner mitochondrial membrane proton gradient, abolishing the ΔΨm. This process eventually oxidative phosphorylation halts, leading to ATP depletion and cellular energy failure (**Figure 3**) (86).

Following mPTP opening and ΔΨm collapse, electron transport chain (ETC) efficiency declines markedly, shifting mitochondrial function toward excessive ROS generation. Calcium-induced mtROS compromises respiratory chain complex integrity and promotes mitochondrial DNA (mtDNA) fragmentation (103). In steatotic hepatocytes, this chronic oxidative stress generates abundant mitochondrial hydrogen peroxide and toxic lipid peroxidation byproducts (11). Evidence from rodent models and *in vitro* cell culture indicates that mtROS exacerbates aberrant intracellular calcium signaling, notably Orai1-mediated calcium pool disruption, and amplifies ER stress. This vicious cycle is proposed to accelerate the organelle failure cascade characteristic of steatohepatitis pathogenesis (48, 104).

During the progression to severe MASH, mitochondrial dysfunction and mtROS overproduction initiate hepatic inflammation (105). Calcium-dependent mitochondrial abnormalities and oxidative stress dictate hepatocyte lipoapoptosis induced by saturated fatty acids including palmitic acid (75, 90). Concurrently, mitochondrial calcium overload activates the ROS/NLRP3 inflammasome axis and drives the cytosolic release of Ox-mtDNA. This process establishes a highly direct mechanistic link between organellar calcium imbalance and innate immune activation. Studies have shown, intracellular calcium overload and the ensuing oxidative stress trigger the persistent opening of mPTP, allowing Ox-mtDNA to leak directly into the cytoplasm (106). Upon entering the cytosol, Ox-mtDNA acts as a potent danger signal that is rapidly sensed by the cyclic GMP-AMP synthase (cGAS), which in turn engages the stimulator of interferon genes (STING) signaling network. The activation of this cGAS-STING pathway in hepatic macrophages serves as a dominant driver of sterile inflammation during liver injury (88). This STING-mediated innate immune response operates in tandem with the ITPR3/Ca2+/NLRP3 axis, violently inducing macrophage pyroptosis and the massive secretion of pro-inflammatory cytokines, a cascading event that critically drives macrophage activation and exacerbates hepatic microenvironmental inflammation (**Figure 3**) (88, 106). Ultrastructurally, upregulated mitochondrial calcium uniporter (MCU) mediates calcium influx and interacts with VDAC1 to induce pathological mitophagy, ultimately precipitating hepatocyte death (87, 107). Therefore, preventing mitochondrial calcium overload restores organellar function and attenuates both mtROS generation and ER stress. This underscores the therapeutic potential of targeting mitochondrial calcium permeability to mitigate lipotoxic liver injury (92).

### 4.3 Pathological rearrangement of the MAMs structural chain

Mitochondria and the ER orchestrate the hepatic metabolic network via specialized physical contact points termed MAMs or mitochondria-ER contact sites (MERCs) (29, 55). Physiologically, MERCs occupy approximately 5% of the hepatocyte mitochondrial surface, providing a structural foundation for efficient inter-organellar coupling (108). In addition, MAMs act as critical conduits coordinating metabolite and lipid shuttling, ROS exchange, and precise transmembrane calcium transport (55, 104). In particular, this calcium flux is mediated by a ternary protein complex enriched at MAMs, where the ER-resident IP3R and mitochondrial outer membrane VDAC1 are physically bridged by the chaperone glucose-regulated protein 75 (Grp75) (39, 108). As a result, maintaining MAM spatial and functional integrity is essential for inter-organellar metabolic communication and cellular homeostasis (104).

However, lipid dyshomeostasis and metabolic stress induce pathological MAM architectural rearrangements (109). Under lipotoxic conditions, lipid overload and saturated fatty acids, notably palmitic acid, hyperactivate and strengthen IP3R-Grp75-VDAC1 complex binding, establishing abnormally tight MERC physical contacts (**Figure 3**) (29). During incipient ER stress, this transient structural coupling serves as a compensatory mechanism to stimulate mitochondrial respiration and bioenergetics (92). Nevertheless, persistent metabolic stress precipitates irreversible MERC reorganization, forcing calcium transport complexes into excessive coupling and disrupting strictly regulated ion gating (36). Consequently, this structural and functional organellar remodeling provides the anatomical basis for pathological ER-to-mitochondria calcium transfer. This drives calcium escape from physiological buffering, resulting in lethal calcium influx into the mitochondrial matrix (36, 109).

The loss of MAM physical integrity and subsequent erroneous signal transduction further precipitate widespread organellar network collapse (104). Under severe ER stress, *in vitro* studies in murine, alongside *in vivo* nutritional models, demonstrate that lipid overload hyperactivates calcium transport complexes and induces aberrant PERK-ALCAT1 complex formation within the MAM domain. This pro-apoptotic complex severely disrupts local calcium homeostasis and impairs ubiquitin-dependent mitophagy, preventing the clearance of damaged mitochondria (**Figure 3**) (89). Driven by pathological MAM reorganization, this lethal disruption of calcium flux and autophagy networks broadly implicates the entire hepatocyte calcium transport machinery, specifically IP3R, Grp75, VDAC1, and MCU. Taken together, this initiates a dual structural and functional collapse of both mitochondria and the ER. This pathology triggers pro-apoptotic signaling and comprehensively impairs hepatic glucose and lipid metabolic networks, representing a core mechanism underlying chronic inflammation and systemic insulin resistance (104). In contrast, murine and cell culture studies suggest that PINK1/Parkin-mediated ubiquitination of the Sigma-1 receptor, a core MAM component, mechanistically inhibits this pathological ER-mitochondria physical contact and excessive calcium transfer, highlighting its therapeutic potential for rescuing organellar integrity (110).

## 5 Calcium signaling determines immune microenvironment remodeling in MASLD

### 5.1 Calcium-dependent inflammatory polarization of Kupffer cells

**Figure 4:**
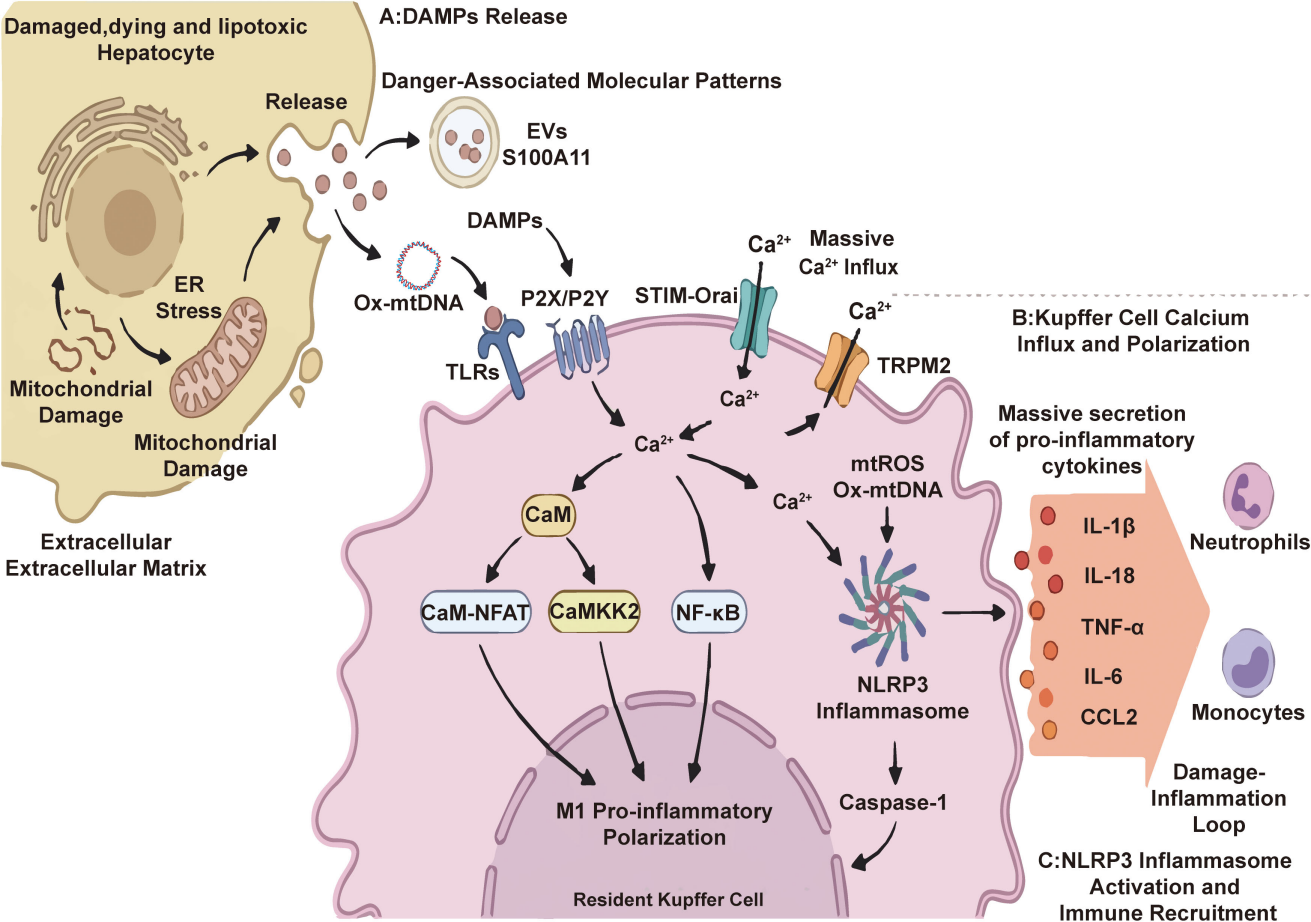
Calcium signaling drives immune microenvironment remodeling and inflammation in MASLD.The hepatic inflammatory cascade is initiated by crosstalk between lipotoxic hepatocytes and the innate immune system, strictly governed by intracellular calcium signaling. **(A)** DAMPs Release: Severe metabolic stress induces hepatocyte organellar damage and cell death, precipitating the extracellular release of DAMPs, prominently including Ox-mtDNA and S100A11-enriched EVs. **(B)** Kupffer Cell Calcium Influx and Polarization: These DAMPs bind to surface receptors on resident Kupffer cells. Receptor engagement, coupled with ER calcium depletion, triggers massive cytosolic calcium influx primarily via the STIM-Orai axis and TRPM2 channels. Elevated cytosolic calcium binds to CaM, activating the CaMKK2 cascade and the CaM-NFAT transcriptional axis. Concurrently, aberrant calcium influx hyperactivates NF-κB. This dual transcriptional reprogramming drives the phenotypic shift toward a pro-inflammatory M1 macrophage. **(C)** NLRP3 Inflammasome Activation and Immune Recruitment: Disrupted calcium homeostasis, alongside mtROS and Ox-mtDNA, serves as a requisite second signal for NLRP3 inflammasome assembly. Activation of the inflammasome induces Caspase-1 cleavage, resulting in the massive secretion of pro-inflammatory cytokines and chemokines. Collectively, this cytokine storm recruits circulating monocytes and neutrophils into the hepatic parenchyma, establishing a self-perpetuating, calcium-dependent damage-inflammation cycle. CaM, Calmodulin; CaMKK2, Calcium/calmodulin-dependent protein kinase kinase 2; CCL2, C-C motif chemokine ligand 2; DAMPs, Damage-associated molecular patterns; ER, Endoplasmic reticulum; EVs, Extracellular vesicles; IL-1β, Interleukin-1 β; IL-6, Interleukin-6; IL-18, Interleukin-18; MASLD, Metabolic dysfunction-associated steatotic liver disease; mtROS, Mitochondrial reactive oxygen species; NFAT, Nuclear factor of activated T-cells; NF-κB, Nuclear factor kappa B; NLRP3, NLR family pyrin domain containing 3; Ox-mtDNA, Oxidized mitochondrial DNA; P2X/P2Y, Purinergic receptors; SOCE, Store-operated calcium entry; STIM/Orai, Stromal interaction molecule/Calcium release-activated calcium channel protein; TLRs, Toll-like receptors; TNF-α, Tumor necrosis factor α; TRPM2, Transient receptor potential cation channel subfamily M member2.

Resident hepatic macrophages, specifically Kupffer cells, orchestrate the hepatic immune cascade by sensing cellular damage signals (1). During metabolic dysfunction and lipid overload, evidence primarily from murine models indicates that damaged hepatocyte-derived signals and pathological cholesterol accumulation activate Kupffer cells, precipitating hepatic inflammation (**Figure 4**) (34). Crucially, experimental studies suggest that intracellular calcium signaling strictly governs this lipotoxic immune activation. *In vitro* and *in vivo* preclinical studies demonstrate that aberrant calcium signaling functions as a primary trigger, compelling immune cells to counter cellular stress. This induces a phenotypic shift toward the pro-inflammatory M1 phenotype, mediating substantial pro-inflammatory cytokine release (3, 17). This process, sustained intracellular calcium elevation dictates specific effector functions and immune cell proliferation during the hepatic innate immune response (44, 51).

In Kupffer cells, rapid intracellular free calcium elevation predominantly originates from SOCE, triggered by ER calcium pool depletion. DAMPs or purinergic signaling via P2Y and P2X receptors precipitate this depletion. Accordingly, this initiates extensive SOCE to supply the requisite calcium for inflammatory polarization (45, 68). Studies have shown, physical coupling between PM Orai channels and the ER calcium sensor STIM mediates this substantial calcium influx. This STIM-Orai signaling axis regulates immune defense mechanisms; thus, *in vitro* studies indicate that pathway hyperactivation or calcium dyshomeostasis induces macrophage organellar stress and subsequent immune hyperactivation (111). As suggested by murine models and *in vitro* experimental data, the non-selective cation channel TRPM2, highly expressed in myeloid macrophages, facilitates calcium signaling that critically regulates pro-inflammatory cytokine release and immune cell chemotactic infiltration (62).

Cytosolic calcium influx, mediated by SOCE and associated cation channels, functions as a secondary messenger activating downstream pro-inflammatory transcriptional networks. Elevated cytosolic calcium initially binds to calmodulin (CaM), initiating a calcium/calmodulin-dependent protein kinase cascade, notably involving CaMKK2, which propagates the macrophage inflammatory response (112). Recent studies also indicate, sustained cytosolic calcium elevation induces CaM dephosphorylation, subsequently activating the nuclear factor of activated T-cells (NFAT) signaling pathway. This CaM-NFAT axis acts as a critical transcriptional switch driving the pro-inflammatory polarization of Kupffer cells, markedly upregulating the secretion of cytokines, notably tumor necrosis factor-α (TNF-α) and interleukin-6 (IL-6) (45, 113). Concurrently, *in vitro* studies using bovine hepatocytes demonstrate that STIM-Orai-mediated aberrant calcium influx hyperactivates the transcription factor NF-κB. This dual NF-κB and NFAT transcriptional activation, driven by cytosolic calcium overload, establishes the Kupffer cell pro-inflammatory phenotype. This cascade precipitates excessive cytokine release, exacerbating the inflammatory cycle within the hepatic pathological microenvironment (111).

### 5.2 Calcium ions and the assembly and activation of the NLRP3 inflammasome

Inflammasomes constitute macromolecular multiprotein complexes widely expressed across parenchymal and non-parenchymal liver cells. Studies have shown, the NOD-like receptor (NLR) family functions as the core pattern recognition receptors, sensing intracellular danger signals to initiate precise complex assembly (114). Based on evidence from murine models, aberrant inflammasome activation extends beyond macrophages, propelling disease cascades in hepatocytes and HSCs. Thus, it acts as a critical cross-amplifying node during MASH progression (115). Under lipotoxic or oxidative stress, *in vitro* studies suggest that aberrant intracellular calcium fluctuations mechanistically link environmental stress to cell death pathways and inflammatory gene expression (116, 117). Experimental animal and cell culture models indicate that disrupted calcium homeostasis, at the cellular level elevated cytosolic free calcium, functions as a requisite upstream second signal triggering NLRP3 inflammasome assembly and subsequent inflammatory cascades in both hepatocytes and macrophages (39, 109).

*In vitro* studies suggest that during lipotoxic stress, synergistic calcium mobilization pathways form a second-signal network that drives inflammasome activation. In murine and cell culture models, prolonged metabolic stress alters the UPR, wherein ER stress-induced calcium leakage directly initiates NLRP3 assembly, thereby precipitating chronic hepatic inflammation and hepatocyte death (36, 97, 109). Multidisciplinary evidence spanning cell culture, murine models, and human MASLD tissues demonstrates that aberrant MAM calcium signaling triggers p-NLRP3(S295)-dependent metabolic inflammation in macrophages, accelerating disease progression (102). Beyond ER calcium release, PM calcium influx proves pivotal. Under metabolic stress, notably hyperglycemia, macrophage surface TRPM2 channels transduce intracellular oxidative stress into inflammatory signals. Mechanistically, TRPM2-mediated calcium influx interacts with p47phox to precisely regulate the aberrant assembly of the TXNIP-NLRP3 inflammasome (59).

Aberrant transmembrane and trans-organellar calcium mobilization facilitates NLRP3 assembly and initiates pyroptosis. *In vitro* studies suggest that excessive ER-to-mitochondria calcium transfer induces mitochondrial calcium overload, triggering ROS accumulation and activating the ROS/NLRP3 signaling axis (88). Conversely, silencing the MCU abrogates ROS production and modulates the MAPKs/NF-κB pathway, effectively attenuating this inflammatory response (87). Crucially, mPTP-mediated release of Ox-mtDNA comprehensively activates the macrophage NLRP3 inflammasome and pyroptosis via pathological ITPR3/Ca2+/NLRP3 signaling axis upregulation (106). Subsequently, inflammasome activation induces caspase-1 cleavage, mediating the maturation and massive secretion of pro-inflammatory cytokines, predominantly IL-1β and IL-18 (109, 114). Ultimately, these abundant danger signals exert a strong chemotactic force, driving peripheral neutrophil and macrophage infiltration into hepatic inflammatory foci, thereby disrupting immune homeostasis within the lipotoxic microenvironment (55, 118).

### 5.3 DAMPs release and amplification of the immune cascade

During MASH pathogenesis, lipotoxic stress and metabolic overload precipitate severe organellar damage and programmed hepatocyte death. Subsequent loss of membrane integrity and impaired autophagy drive the extracellular release of undegraded organelles and immunogenic intracellular contents, generating DAMPs (65, 119). Consequently, stressed and senescent hepatocytes act as pivotal nodes, linking parenchymal injury to chronic immune-mediated inflammation via these endogenous danger signals (38, 120). In particular, studies utilizing rat models of diet-induced NAFLD demonstrate that mtDNA extruded from damaged mitochondria functions as a primary DAMP, profoundly expanding the local danger signal matrix (103). Concurrently, preclinical studies suggest that lipotoxic steatotic hepatocytes secrete abundant extracellular vesicles (EVs) enriched in specific alarmins, notably S100A11. These EVs function as high-frequency microenvironmental danger signals, propagating lipotoxic information across hepatic intercellular communication networks (25, 121–123). Emerging evidence further indicates that calcium dysregulation is not merely a downstream consequence of hepatocyte injury but also an active regulator of extracellular vesicle biogenesis. Sustained elevation of cytosolic Ca^2+^ promotes plasma membrane remodeling and vesicle shedding, while simultaneously influencing the selective loading of stress-associated cargo into EVs. Under lipotoxic conditions, ER stress markedly increases S100A11 expression and enriches its packaging into hepatocyte-derived EVs (121). Likewise, lipotoxic hepatocytes exhibit enhanced release of small extracellular vesicles carrying LIMA1, which promotes hepatic stellate cell activation by suppressing PINK1–Parkin-dependent mitophagy in recipient cells (123). These findings suggest that calcium-dependent EV biogenesis provides a mechanistic link between intracellular calcium dyshomeostasis and the propagation of inflammatory and profibrotic signals within the hepatic microenvironment.

Microenvironmental DAMPs and their vesicular carriers rapidly surpass local physiological thresholds, directly targeting and activating adjacent quiescent immune cells (124). Preclinical studies demonstrate that these concentrated extracellular mediators bind pattern recognition receptors, notably Toll-like receptors (TLRs), on resident macrophages and infiltrating immune cells, eliciting aberrant intracellular signal transduction (125). Crucially, investigations in murine models indicate that this receptor engagement stimulates and amplifies target cell calcium responses, functioning as an upstream molecular switch that drives cellular polarization and metabolic inflammatory transcriptional reprogramming (38, 119). Driven by calcium channel hyperactivation and resultant calcium influx, *in vitro* studies suggest that the macrophage network undergoes an irreversible phenotypic shift. This comprehensively activates a broad tissue-spanning cellular circuit encompassing resident Kupffer cells, sinusoidal endothelial cells, and bone marrow-derived immune populations (124, 126).

Under this calcium-dependent activation framework, local innate immune defenses transition into a highly destructive, pro-inflammatory positive feedback loop (105). Massive DAMP exposure hyperactivates Kupffer cells, propelling the robust secretion of complex pro-inflammatory cytokine and chemokine networks, predominantly tumor necrosis TNF-α, IL-1β and CCL2 (7, 118). Subsequently, these pathogenic chemotactic gradients continuously recruit circulating monocytes and pro-inflammatory adaptive immune cells across the vascular endothelium into hepatic lesions, accelerating tissue microenvironmental degradation. As a result, this cascade—initiated by receptor activation, propagated by intracellular calcium dyshomeostasis, and culminating in excessive cytokine release—establishes a self-perpetuating damage-inflammation cycle within the hepatic parenchyma (**Figure 4**) (118).

## 6 Downstream fibrotic processes driven by immune-metabolic feedback loops

### 6.1 Activation of HSCs by inflammatory mediators

Liver fibrosis, a hallmark of progressive diseases including MASH, is characterized by excessive extracellular matrix (ECM) deposition and architectural distortion (127, 128). HSCs are the primary drivers of this fibrotic remodeling (129). During the transition from inflammation to fibrosis, amplified metabolic stress, lipotoxicity, and parenchymal-non-parenchymal cross-talk establish a pro-fibrotic niche (130, 131). Consequently, this multicellular communication disrupts immunostasis, perpetuating innate immune activation. The subsequent paracrine release of inflammatory mediators stimulates quiescent HSCs to transdifferentiate into proliferative, contractile myofibroblasts (124, 132).

Among these mediators, transforming growth TGF-β derived from activated Kupffer cells primarily orchestrates HSC transdifferentiation (1, 125). In hepatocytes, TGF-β stimulates HSC activation, contraction, and pro-fibrotic gene expression via the canonical SMAD signaling pathway (118, 133). Concurrently, macrophages and LSECs secrete mediators including tissue inhibitor of metalloproteinases 1 (TIMP1), IL-1β and NLRP3 inflammasome signals. These factors synergistically induce HSCs to synthesize abundant α-smooth muscle actin (α-SMA) and type I collagen, thereby driving progressive fibrosis (134, 135). Furthermore, this immune-HSC crosstalk is bidirectional. Receptors including P2X7R and distinct chemokines consolidate pathological feedback loops between macrophages and HSCs. Activated HSCs subsequently induce phenotypic shifts in surrounding monocyte-derived macrophages, creating a positive-feedback loop that accelerates fibrotic remodeling (11, 14, 136).

Beyond innate immune signaling, DAMPs from injured hepatocytes serve as critical upstream triggers for HSC activation. Lipotoxicity and organelle stress prompt steatotic hepatocytes to release soluble pro-fibrotic factors (115, 137). For instance, stress-damaged hepatocytes overexpressing E4BP4 secrete paracrine mediators including osteopontin (OPN) and lipocalin-2 (LCN2) to directly activate adjacent HSCs (138, 139). Additionally, hepatocyte-derived extracellular vesicles (EVs) containing alarmins including LIMA1 deliver fibrogenic constituents and suppress mitochondrial autophagy in HSCs, thereby accelerating their fibrotic transformation (122, 123). Amidst these inflammatory and injury signals, mitogens namely platelet-derived growth factor (PDGF) stimulate HSC proliferation via the ERK-MAPK and PI3K-Akt pathways. Ultimately, enhanced intracellular autophagy and transcriptional reprogramming mediated by factors including JUNB and SMAD satisfy the energy demands of activated HSCs, culminating in severe stromal deposition and hepatic remodeling (100, 118, 130, 140).

### 6.2 The decisive role of calcium channels in HSC activation and contraction

Liver fibrosis represents the terminal stage of persistent hepatic injury and dictates the progression from simple steatosis to MASH (124). During this process, HSC transdifferentiation into myofibroblasts requires metabolic reprogramming analogous to the Warburg effect (141). Although inhibiting glycolysis and glutamine catabolism attenuates HSC anabolism, the fundamental prerequisite for extensive ECM production and cellular contraction is elevated cytoplasmic calcium concentration ([Ca^2+^]cyt) alongside altered calcium flux kinetics (39, 142, 143). Therefore, dysregulated calcium channels and intracellular calcium regulatory networks contribute substantially to the pathogenic transformation of HSCs, highlighting calcium homeostasis as an important therapeutic target for limiting fibrogenesis (144, 145).

HSC activation relies profoundly on extracellular calcium influx via specific transmembrane channels. Notably, the TRP channel family, including TRPC6 and TRPV4, mediates this requisite calcium influx to drive myofibroblast transdifferentiation (56, 64). Concurrently, aberrant PI3K/Akt signaling translocates acid-sensing ion channel 1a (ASIC1a) to the cell membrane. This pathological calcium entry provides essential survival signals for HSC activation and enhances cellular migration via calcium-dependent autophagy (100, 146). Meanwhile, the CaSR is overexpressed in α-SMA-positive activated HSCs, whereby marginal microenvironmental extracellular calcium increments trigger profound intracellular calcium surges (143). Additionally, paracrine signaling from dysfunctional LSECs alters HSC calcium sensitivity, exacerbating ECM deposition and network remodeling (66, 134).

Aberrant intracellular calcium elevation triggers extensive downstream signaling cascades and organelle remodeling. At the cellular level, profound calcium influx activates the CaM/CaMKII pathway, perpetuating HSC proliferation. Consequently, fibrogenic factors including PDGF, angiotensin II, and activin A strictly depend on this intracellular calcium burst to induce HSC migration and proliferation; conversely, intracellular calcium chelators notably BAPTA-AM substantially attenuate these processes (147–149). At the organelle level, disrupted ER calcium homeostasis serves as a primary endogenous signal for HSC phenotypic deterioration. This disruption, coupled with SOCE, induces sustained free calcium release to drive cellular contraction and matrix secretion (81). Furthermore, calcium signaling induces severe ER stress via the Calpain-2 pathway (150). Concurrently, mitochondrial calcium uptake regulates actin cytoskeleton reorganization during pathogenic HSC migration. Importantly, the biological consequence of targeting the MCU is profoundly cell-type specific across MASH progression. While continuous basal calcium flux remains indispensable for parenchymal hepatocytes to maintain AMPK-dependent lipid homeostasis and prevent early steatosis, non-parenchymal cellular networks exhibit a fundamentally distinct signaling dependency during fibrogenesis. In hepatocytes, activated HSCs heavily rely on this massive MCU-mediated calcium influx to fuel actin reorganization and pathological ECM secretion. *In vivo*, targeted knockdown of the MCU suppresses early-stage inflammation and attenuates MASH-associated downstream fibrosis (87, 147). Modulating calcium ionophores including A23187 reshapes the fate of TGF-β1-stimulated HSCs, supporting the concept that aberrant calcium influx and organellar calcium remodeling contribute to the initiation and maintenance of liver fibrosis (116).

## 7 Pharmacological interventions and clinical translation targeting calcium signaling and immune network

### 7.1 The ‘hidden’ calcium-regulating effects of existing metabolic and immunomodulatory drugs

Following the approval of the thyroid hormone receptor agonist resmetirom and the clinical success of glucagon-like peptide-1 receptor agonists (GLP-1RAs), the pharmacological management of MASH has significantly advanced (151). Beyond attenuating hepatic TG accumulation and restoring systemic insulin sensitivity, the hepatoprotective efficacy of these metabolic modulators fundamentally depends on restoring intracellular organelle homeostasis (12). Mechanistically, these therapeutics converge on adenosine monophosphate-activated protein kinase (AMPK), the central hub of hepatic energy metabolism. Consequently, systemic intervention reverses lipid-induced microenvironmental collapse. Studies have shown, alleviating ER stress and re-establishing intracellular calcium homeostasis are fundamental to arresting pathological progression (12, 152).

In addition, GLP-1 analogs and sodium-glucose cotransporter 2 (SGLT2) inhibitors exhibit substantial potential for organelle-targeted intervention alongside correcting systemic metabolic dysregulation. Notably, GLP-1 analogs including exenatide reverse lipid-induced inhibition of hepatic ER calcium release. This mechanism relies on GLP-1 receptor-mediated cAMP signaling to normalize intracellular calcium flux and regulate ER calcium mobilization (53, 73). Additionally, exendin-4 reverses aberrant SOCE in steatotic hepatocytes and mitigates severe ER stress via specific SIRT1 pathway activation. Similarly, SGLT2 inhibitors restore hepatocyte calcium homeostasis. By activating the AMPK, SIRT1, and PPARγ co-activator 1α networks, these agents stimulate mitochondrial lipid oxidation and reparative autophagy, thereby fundamentally inhibiting DNL and remodeling organelle homeostasis (153).

Moreover, nuclear receptor agonists exhibit significant calcium-regulatory dependency during intervention against inflammatory cascades and organelle damage (154). Specifically, the farnesoid X receptor (FXR) agonist obeticholic acid profoundly inhibits ER stress by inducing transcription of the SERCA2 (77). Concurrently, FXR agonists regulate intracellular calcium homeostasis and attenuate inflammatory responses by inhibiting ER stress-mediated NLRP3 inflammasome activation, simultaneously improving lipid metabolism (109). Natural compounds including betulinic acid similarly depend on FXR pathway activation to alleviate ER stress (155). In contrast, the pan-PPAR agonist lanifibranor has emerged as a leading pharmacological candidate for NAFLD (156, 157).

Pharmacological restoration of SERCA transport activity effectively curbs ER stress and corrects downstream mitochondrial dysfunction, representing a critical mechanism for limiting diet-induced steatohepatitis progression (75). Numerous metabolic protectants and natural compounds target this pathway. For instance, the natural antioxidant chrysanthemum extract upregulates SERCA2b expression to ameliorate insulin resistance, whereas the anti-inflammatory mediator Maresin 1 inhibits ER stress-driven hepatic lipogenesis via AMPK/SERCA2b pathway activation (13, 83). Additionally, the metabolic modulator taurocholic acid profoundly inhibits the ER stress network, effectively blocking subsequent pro-apoptotic caspase-12 activation (116, 156). Recent studies also indicate, diverse agents including autophagy-targeting mTOR inhibitors, oxidative stress-alleviating S-adenosylmethionine, and ER calcium channel-blocking dantrolene interrupt hepatocyte apoptosis triggered by compensatory calcium refilling or overload. Overall, these findings suggest that restoring calcium homeostasis represents an important component of the hepatoprotective mechanisms of several existing metabolic therapies (76, 119, 158).

### 7.2 Preclinical exploration of targeted calcium channel modulators

Given the lack of direct therapeutics for MASH, modulating intracellular calcium flux via channel blockade represents an urgent strategy to arrest organelle dysfunction and lipotoxicity at their source (14). Preclinical studies utilizing high-fidelity human *in vitro* systems, including hiPSC-derived hepatocytes, 3D perfusion models, and precision-cut liver slices, robustly validate the efficacy of drugs targeting ER stress and calcium dysregulation (28, 159). Experimental evidence indicates, targeted calcium channel intervention effectively alters the pathological trajectory in MASLD animal models. For instance, CaSR antagonists attenuate extracellular calcium influx-induced cytoplasmic calcium overload in HSCs, providing a viable anti-fibrotic pharmacological target (143). Furthermore, the microenvironmental stiffness-activated Piezo1 mechanosensitive channel and aberrantly expressed ASIC1a have emerged as novel targets for impeding lipid metabolic reprogramming, fibrosis progression, and HCC malignant transformation (69, 146, 160).

Within the ER-cytoplasmic calcium exchange network, specific inhibitors exhibit substantial potential to abrogate malignant signaling cascades. Notably, novel small-molecule inhibitors targeting the IP3R directly antagonize HSC activation in 3D human liver organoids, presenting a promising strategy to reverse severe hepatic fibrosis (144). *In vitro* IP3R or store-operated calcium channel (SOCC) blockade effectively attenuates lipotoxicity-induced cytoplasmic calcium overload and subsequent apoptosis (54). Concurrently, CaM 2 functions as a negative regulator to suppress excessive SOCE activation (45). Additionally, calcium-specific chelators including BAPTA-AM completely abolish fatty acid-induced apoptotic stress and restore normal oxygen consumption (90, 161). Regarding downstream signaling, specifically inhibiting the calcium-dependent kinase CaMKK2 with the selective antagonist STO-609 effectively reverses pathological states across *in vivo* and *in vitro* models (112).

Significant progress has occurred in targeting TRP channels and MCUs, despite inherent toxicity challenges. Mechanistically, targeted inhibition of TRPM2 or its downstream CaMKII pathway blocks NLRP3 inflammasome activation and induces autophagy to alleviate hepatic injury; for example, the natural compound curcumin inhibits oxidative stress-induced TRPM2 activation (59, 62, 63). Additionally, novel TRPC6 inhibitors effectively reduce inflammation and fibrosis in animal models (64). However, systemic TRP channel interventions necessitate caution due to potential toxicity. Clinical evidence indicates that specific TRPV1 blockade induces severe systemic adverse effects including hyperthermia, warranting stringent safety considerations (57). To circumvent these effects, advanced localized delivery utilizing supramolecular hydrogel patches containing the calcium channel blocker verapamil achieves targeted detoxification, induces autophagy, and ameliorates obesity-induced MASLD (17, 162). At the mitochondrial level, specific MCU inhibition via small interfering RNA or chemical inhibitors including Ru360 prevents excessive mitophagy and rescues organellar dysfunction. Concurrently, cyclosporine A-mediated inhibition of the mPTP prevents Ox-mtDNA leakage, thereby rescuing macrophages from inflammatory pyroptosis (86, 106, 107).

Strategies directly restoring ER calcium homeostasis primarily focus on specific SERCA activators. In contrast to destructive cell death induced by specific SERCA inhibition, small-molecule activators including CDN1163 specifically reactivate SERCA to successfully attenuate liver fibrosis and microenvironmental inflammation (39, 75, 83). Furthermore, urolithin A enhances hepatocyte survival by directly binding SERCA, whereas the novel FXR agonist Atractylolide II reduces ER stress by stimulating the FXR-SERCA2-eIF2α axis (77, 85). Endogenous protective factors including hepatic stimulator substance (HSS) effectively counteract free fatty acid-induced apoptosis by enhancing SERCA activity (16). Targeting the SERCA axis to restore ER calcium homeostasis represents a promising preclinical strategy that warrants further validation in clinically relevant settings for combating hepatic and cardiovascular metabolic diseases (81). The key regulatory mechanisms, core molecular targets, and their preclinical significance in modulating calcium signaling are summarized in **Table 1**.

**Table 1:** Key pharmacological targets and regulatory mechanisms of calcium signaling in hepatic metabolic diseases.

Functional module & localization	Targets/factors	Regulatory mechanism	Core molecular mechanism	Pathological/physiological significance	Reference
ER Homeostasis & Stress	IP3R & SOCCs	Inhibition/Blockade	Blocks ER calcium release and subsequent extracellular influx, preventing cytoplasmic and mitochondrial calcium overload	Attenuates hepatocyte damage and heavy metal-induced apoptosis; therapeutic strategy for NAFLD	(39, 54)
	SERCA Isoforms (SERCA/SERCA2)	Activation/Activity restoration	Scavenges ROS and mediates eIF2α dephosphorylation to restore ER homeostasis, downregulating UPR markers	Protects hepatocytes from FFA-induced lipoapoptosis; mitigates NAFLD and obesity-associated metabolic dysfunction	(16, 77)
PM & Inflammasome	L-type Channels & TRPM2	Inhibition/Downregulation	Activates autophagic flux to specifically suppress the TXNIP/NLRP3 inflammasome pathway	Alleviates MASLD/MAFLD progression, insulin resistance, and hepatic I/R injury	(17, 59)
	CRAC (Orai1/STIM1)	Activation (SOCE)	STIM1 senses ER calcium depletion and couples with Orai1 to trigger massive calcium influx, activating NFAT nuclear translocation	Critical for T-cell development, cytokine production, and inflammatory disease modulation	(45)
	TRPC6	Inhibition	Prevents Ca^2+^ and Na^+^ influx that directly activates intracellular pathways associated with inflammation and fibrosis	Potential therapeutic target for combating liver fibrosis, as well as kidney and lung diseases	(64)
Mitochondrial Transport	MCU	Inhibition/Genetic deletion	Prevents mitochondrial Fe^2+^/Ca^2+^ uptake and subsequent oxidative/nitrative stress	Prevents APAP-induced hepatotoxicity	(86)
Microenvironmental Sensors & Fibrosis	Piezo1/CaSR/ASIC1a	Activation/Regulation/Antagonism	Senses specific microenvironmental stimuli to trigger intracellular Ca^2+^, YAP, or CaM/CaMKII signaling cascades	Collectively drives HSC activation, proliferation, and early-stage liver fibrogenesis	(143, 146, 160)

APAP, Acetaminophen; ASIC1a, Acid-sensing ion channel 1a; CaM, Calmodulin; CaMKII, Calcium/calmodulin-dependent protein kinase II; CaSR, Calcium-sensing receptor; CRAC, Calcium release-activated calcium channel; eIF2α, Eukaryotic initiation factor 2 α; ER, Endoplasmic reticulum; FFA, Free fatty acid; HSC, Hepatic stellate cell; I/R, Ischemia/reperfusion; IP3R, Inositol 1, 4,5-trisphosphate receptor; MAFLD, Metabolic dysfunction-associated fatty liver disease; MASLD, Metabolic dysfunction-associated steatotic liver disease; MCU, Mitochondrial calcium uniporter; NAFLD, Non-alcoholic fatty liver disease; NFAT, Nuclear factor of activated T-cells; NLRP3, NLR family pyrin domain containing 3; ROS, Reactive oxygen species; SERCA, Sarco/endoplasmic reticulum Ca^2+^-ATPase; SOCCs, Store-operated calcium channels; SOCE, Store-operated calcium entry; STIM1, Stromal interaction molecule 1; TRPC6, Transient receptor potential canonical 6; TRPM2, Transient receptor potential melastatin 2; TXNIP, Thioredoxin-interacting protein; UPR, Unfolded protein response; YAP, Yes-associated protein.

### 7.3 Future prospects for combined calcium channel and immune-targeted therapies

Monotherapies for NASH primarily target isolated metabolic or fibrotic pathways, fundamentally limiting their efficacy against irreversible progression to cirrhosis (154). Consequently, combating the complex pro-fibrotic microenvironment necessitates multi-targeted therapies to disrupt pathological crosstalk among hepatocytes, macrophages, and HSCs (124, 137). In particular, synergizing peroxisome proliferator-activated receptor (PPAR) agonists with antioxidants significantly improves survival amidst advanced hepatic injury (158). Importantly, in NASH-associated HCC, where protein kinase inhibitor monotherapy exhibits profound toxicity and limited efficacy, systemic metabolic remodeling is imperative. Ultimately, simultaneously targeting metabolic and organellar stress networks offers a robust strategy to arrest irreversible disease progression (163).

Integrating calcium homeostasis restoration with metabolic and immune interventions constitutes a frontier in combination therapy. Notably, the CaSR emerges as a critical therapeutic target for hepatic metabolic disorders owing to its central mechanistic role in both networks (67). Synergizing anti-inflammatory agents including tumour necrosis factor-α monoclonal antibodies with calcium modulators effectively impedes the progression from MASH to HCC (3). Additionally, combining the systemic immunomodulatory properties of vitamin D with the cytoprotective effects of calcium channel blockers provides a dual-pronged strategy against MASH and severe fibrosis (145). To precisely disrupt intercellular crosstalk, employing neutralizing antibodies or gene silencing against OPN effectively abrogates the hepatocyte E4BP4 axis-driven fibrotic transformation of HSCs, thereby substantiating the translational viability of multi-target interventions (138).

Mesenchymal stem cell-derived extracellular vesicles (MSC-EVs) have recently emerged as a promising cell-free therapeutic strategy for MASLD. Preclinical studies indicate that adipose-derived MSC-EVs carrying miR-223-3p can attenuate steatosis and fibrosis in murine NAFLD models through suppression of E2F1 signaling (164). Likewise, MSC-derived small extracellular vesicles have been shown to reduce hepatic lipid accumulation *in vitro* and in high-fat diet-induced mouse models by limiting DRP1-mediated mitochondrial fission and improving mitochondrial homeostasis (165). Given the close interplay between Ca^2+^ signaling, mitochondrial dynamics, and immune activation, MSC-EVs may complement calcium-targeted therapies by simultaneously restoring organelle function and modulating hepatic inflammation. In addition, bioengineered mitochondria-enriched extracellular vesicles generated through activation of the CD38/IP3R/Ca^2+^ pathway provide a potential framework for future organelle-based therapeutic approaches (166). Although these findings remain largely preclinical, they highlight the potential value of combining MSC-EV-based strategies with calcium channel and immune-targeted interventions in MASLD.

To deploy these multi-target therapies while mitigating systemic toxicity, advanced targeted delivery systems are imperative. Specifically, amphiphilic block copolymer nanoparticles including micelles and polymeric vesicles provide efficient platforms for targeting macrophage metabolism and ion channels (167). Furthermore, modifying liposomes with PDGFR-β-targeting antibodies facilitates the precise delivery of anti-fibrotic or ion channel-blocking agents directly to activated HSCs, substantially improving safety profiles. In parallel, phosphatidylserine-enriched liposomes selectively target Kupffer cells and liver-resident macrophages to reprogram the local immune microenvironment (101). Additionally, inflammatory-responsive biomimetic nanoparticles exhibit substantial potential for targeted anti-fibrotic therapy by precisely modulating the ER stress network (168). Nanoparticle clusters capable of dynamic morphological adaptations, alongside rigid-flexible hybrid biomimetic nanoemulsions, efficiently penetrate microenvironmental barriers. Consequently, these advanced nanotherapeutics hold immense translational potential for enhancing the precision and efficacy of treatments for hepatic metabolic diseases (169). Despite the encouraging therapeutic potential of calcium-targeted interventions, their clinical translation requires careful consideration of safety and tissue specificity. Because calcium signaling is a ubiquitous second messenger involved in numerous physiological processes, indiscriminate modulation of calcium channels, pumps, or transporters may produce unintended effects beyond the liver (170). For example, prolonged activation of SERCA or inhibition of IP3Rs could disturb physiological calcium homeostasis in excitable tissues, including the nervous system, whereas excessive suppression of mitochondrial calcium uptake through MCU inhibition may impair mitochondrial metabolism and immune cell function (170, 171). Therefore, future development of calcium-targeted therapies should prioritize liver-specific delivery, cell-selective targeting, and rigorous safety evaluation to maximize therapeutic efficacy while minimizing off-target toxicity before clinical translation (172).

While these emerging strategies offer considerable therapeutic promise, several important limitations and translational challenges should also be acknowledged. Most mechanistic insights summarized in this review are derived from rodent models and *in vitro* studies, whereas direct evidence from human liver tissues and prospective clinical investigations remains relatively limited. To provide readers with a clearer overview of the current evidence landscape, the primary experimental evidence supporting the major calcium-dependent mechanisms discussed in this review is summarized in **Table 2**. Future studies integrating human multi-omics datasets, spatial transcriptomics, and well-designed clinical cohorts will be essential to validate these mechanisms and identify clinically actionable calcium-related therapeutic targets.

**Table 2:** Summary of the major calcium-dependent mechanisms in MASLD and the primary experimental evidence supporting each mechanism.

Calcium-dependent mechanism	Representative finding	Primary evidence source	References
Plasma membrane calcium influx	Dysregulated calcium channels promote hepatic calcium overload and metabolic dysfunction	Human studies, animal models and cell culture	(43, 55, 67)
ER calcium dyshomeostasis	SERCA dysfunction and ER calcium depletion trigger ER stress and UPR activation	Animal models and cell culture	(75, 77)
Mitochondrial calcium overload	Excessive mitochondrial Ca^2+^ uptake induces mtROS production and Ox-mtDNA release	Animal models and cell culture	(86, 88, 90)
MAM remodeling	Impaired ER–mitochondria calcium transfer aggravates organelle dysfunction	Animal models and cell culture	(85, 91, 94)
Kupffer cell activation	Calcium signaling promotes macrophage inflammatory polarization	Animal models and cell culture	(97, 102, 105)
NLRP3 inflammasome activation	Calcium overload drives inflammasome activation and inflammatory cytokine release	Human studies, animal models and cell culture	(106, 109, 118)
DAMPs/EVs-mediated immune amplification	DAMPs and hepatocyte-derived EVs amplify inflammatory signaling inflammasome assembly	Animal models and cell culture	(25, 121, 125)
HSCs activation and fibrosis	Calcium-dependent signaling promotes HSCs activation and liver fibrosis	Human studies, animal models and cell culture	(138, 143, 145)

DAMPs, Damage-associated molecular patterns; ER, Endoplasmic reticulum; EVs, Extracellular vesicles; HSCs, Hepatic stellate cells; MAM, Mitochondria-associated membrane; MASLD, Metabolic dysfunction-associated steatotic liver disease; mtROS, Mitochondrial reactive oxygen species; NLRP3, NLR family pyrin domain containing 3; Ox-mtDNA, Oxidized mitochondrial DNA; SERCA, Sarco/endoplasmic reticulum Ca^2+^-ATPase; UPR, Unfolded protein response.

## 8 Conclusion

In conclusion, aberrant intracellular calcium signaling is not merely a bystander in lipotoxicity, but a fundamental pathogenic driver that governs the transition from isolated steatosis to MASH and progressive liver fibrosis. The catastrophic cascade—initiated by membrane biophysical degradation, propagated by ER-mitochondrial calcium transport collapse, and amplified by profound immune network remodeling—is intrinsically tethered to calcium dyshomeostasis. The terminal fibrotic transdifferentiation of HSCs is strictly dependent on these calcium-mediated inflammatory and metabolic feedback loops. Therefore, effectively combating MASLD necessitates a paradigm shift from mono-therapeutic approaches to comprehensive interventions. Future clinical and translational efforts must prioritize multi-targeted strategies that simultaneously restore intra-organellar calcium homeostasis, resolve organelle stress, and disrupt pathological immune-fibrotic crosstalk. Leveraging advanced biomimetic and targeted nanodelivery platforms to deploy these calcium-modulating agents will be crucial to maximizing hepatoprotective efficacy while mitigating systemic toxicity.
